# 1,4-Di­bromo-2,5-di-*p*-toluoyl­benzene

**DOI:** 10.1107/S160053681302237X

**Published:** 2013-08-17

**Authors:** Guang-Ke He, Guang-Liang Song, Chen Chen, Hong-Jun Zhu

**Affiliations:** a Department of Applied Chemistry, College of Science, Nanjing University of Technology, Nanjing 210009, People’s Republic of China

## Abstract

In the title compound, C_22_H_16_Br_2_O_2_, which has approximate non-crystallographic inversion symmetry, the dihedral angles between the central ring and the pendant rings are 89.1 (4) and 82.4 (3)°; the dihedral angle between the pendant rings is 12.1 (4)°. In the crystal, the packing is influenced by van der Waals forces and no aromatic π–π stacking is observed.

## Related literature

For background to the applications of the title compound, see: Shimizu *et al.* (2011 ▶). For further synthetic details, see: Chardonnens & Salamin (1968 ▶).
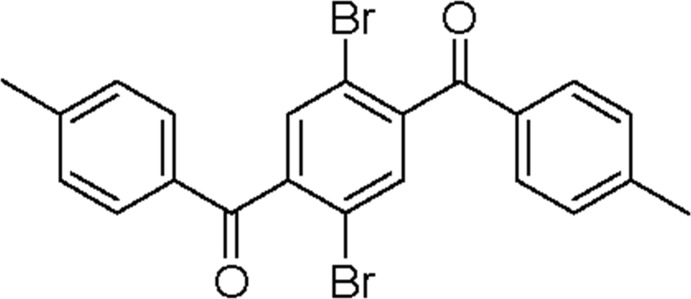


## Experimental

### 

#### Crystal data

C_22_H_16_Br_2_O_2_
*M* *_r_* = 472.17
Monoclinic, 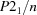

*a* = 9.855 (2) Å
*b* = 12.064 (2) Å
*c* = 16.345 (3) Å
β = 97.61 (3)°
*V* = 1926.2 (7) Å^3^
*Z* = 4
Mo *K*α radiation
μ = 4.22 mm^−1^
*T* = 293 K
0.20 × 0.10 × 0.10 mm

#### Data collection

Enraf–Nonius CAD-4 diffractometer
Absorption correction: ψ scan (North *et al.*, 1968 ▶) *T*_min_ = 0.486, *T*_max_ = 0.678
3664 measured reflections
3452 independent reflections
1518 reflections with *I* > 2σ(*I*)
*R*_int_ = 0.054
3 standard reflections every 200 reflections intensity decay: none

#### Refinement

*R*[*F* ^2^ > 2σ(*F* ^2^)] = 0.065
*wR*(*F* ^2^) = 0.133
*S* = 1.00
3452 reflections
235 parameters
48 restraints
H atoms treated by a mixture of independent and constrained refinement
Δρ_max_ = 0.46 e Å^−3^
Δρ_min_ = −0.40 e Å^−3^

### 

Data collection: *CAD-4 EXPRESS* (Enraf–Nonius, 1994 ▶); cell refinement: *CAD-4 EXPRESS*; data reduction: *XCAD4* (Harms & Wocadlo, 1995 ▶); program(s) used to solve structure: *SHELXS97* (Sheldrick, 2008 ▶); program(s) used to refine structure: *SHELXL97* (Sheldrick, 2008 ▶); molecular graphics: *SHELXTL* (Sheldrick, 2008 ▶); software used to prepare material for publication: *SHELXTL*.

## Supplementary Material

Crystal structure: contains datablock(s) I, s. DOI: 10.1107/S160053681302237X/hb7121sup1.cif

Structure factors: contains datablock(s) I. DOI: 10.1107/S160053681302237X/hb7121Isup2.hkl

Supplementary material file. DOI: 10.1107/S160053681302237X/hb7121Isup3.cml

Additional supplementary materials:  crystallographic information; 3D view; checkCIF report

## Figures and Tables

**Fig. 1.**
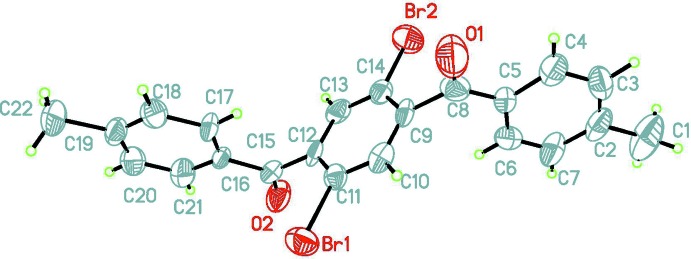
The molecular structure of (I), with displacement ellipsoids drawn at the 50% probability level.

**Fig. 2.**
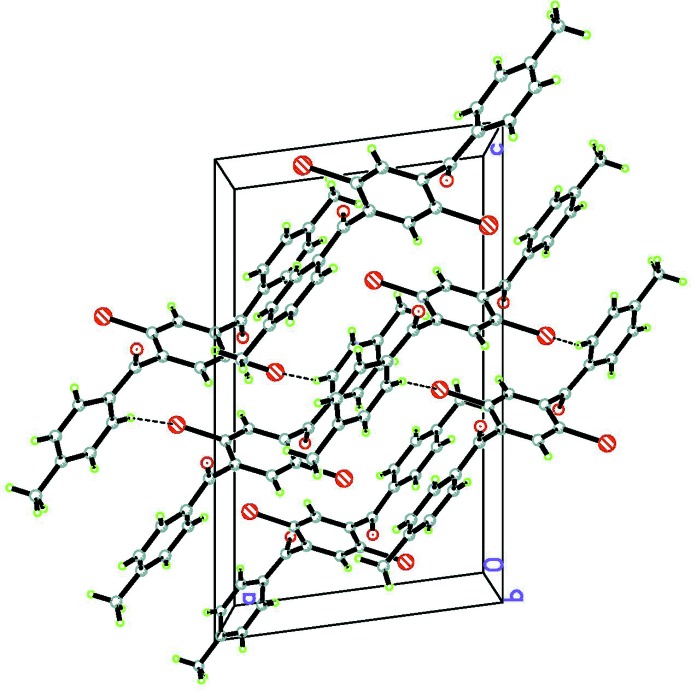
A packing diagram of (I).

## supplementary crystallographic information

### 1. Comment

The title compound, 1,4-dibromo-2,5-di-*p*-toluoylbenzene is an important intermediate not only for manufacturing OLED materials, but also for sensing and switching devices that utilize solid-state luminescence as an output. (Shimizu *et al.*, 2011). We now report here its the crystal structure.

The molecular structure of (I) is shown in Fig. 1, and the selected geometric parameters are give in Table 1. The dihedral angles between the phenyl rings in *p*-toluoyl and the dibromobenzene are 89.14 (2)° and 82.41 (2)°. The phenyl rings are almost parallel with the maximum deviation of 0.26 (8)°. The crystal packing of the molecules in the crystal is influenced by van der Waals forces.

### 2. Experimental

The title compund was synthesized according to the published procedure (Chardonnens & Salamin, 1968). Colourless blocks were obtained by dissolving it (0.5 g) in tetrahydrofuran (20 ml) and evaporating the solvent slowly at room temperature for about 10 d.

### 3. Refinement

H atoms were positioned geometrically and refined as riding groups, with C—H = 0.93 Å for aromatic H, and constrained to ride on their parent atoms, with *U*_iso_(H) = *xU*_eq_(C), where *x* = 1.2 for aromatic H, and *x* = 1.5 for other H.

### Crystal data

**Table:**

C_22_H_16_Br_2_O_2_	*F*(000) = 936
*M**_r_* = 472.17	*D*_x_ = 1.628 Mg m^−^^3^
Monoclinic, *P*2_1_/*n*	Mo *K*α radiation, λ = 0.71073 Å
Hall symbol: -P 2yn	Cell parameters from 25 reflections
*a* = 9.855 (2) Å	θ = 10–13°
*b* = 12.064 (2) Å	µ = 4.22 mm^−^^1^
*c* = 16.345 (3) Å	*T* = 293 K
β = 97.61 (3)°	Block, colourless
*V* = 1926.2 (7) Å^3^	0.20 × 0.10 × 0.10 mm
*Z* = 4	

### Data collection

**Table:**

Enraf–Nonius CAD-4 diffractometer	1518 reflections with *I* > 2σ(*I*)
Radiation source: fine-focus sealed tube	*R*_int_ = 0.054
Graphite monochromator	θ_max_ = 25.2°, θ_min_ = 2.1°
ω/2θ scans	*h* = −11→11
Absorption correction: ψ scan (North *et al.*, 1968)	*k* = 0→14
*T*_min_ = 0.486, *T*_max_ = 0.678	*l* = 0→19
3664 measured reflections	3 standard reflections every 200 reflections
3452 independent reflections	intensity decay: none

### Refinement

**Table:**

Refinement on *F*^2^	Primary atom site location: structure-invariant direct methods
Least-squares matrix: full	Secondary atom site location: difference Fourier map
*R*[*F*^2^ > 2σ(*F*^2^)] = 0.065	Hydrogen site location: inferred from neighbouring sites
*wR*(*F*^2^) = 0.133	H atoms treated by a mixture of independent and constrained refinement
*S* = 1.00	*w* = 1/[σ^2^(*F*_o_^2^) + (0.050*P*)^2^] where *P* = (*F*_o_^2^ + 2*F*_c_^2^)/3
3452 reflections	(Δ/σ)_max_ < 0.001
235 parameters	Δρ_max_ = 0.46 e Å^−^^3^
48 restraints	Δρ_min_ = −0.40 e Å^−^^3^

### Special details

**Table:**

Geometry. All e.s.d.'s (except the e.s.d. in the dihedral angle between two l.s. planes) are estimated using the full covariance matrix. The cell e.s.d.'s are taken into account individually in the estimation of e.s.d.'s in distances, angles and torsion angles; correlations between e.s.d.'s in cell parameters are only used when they are defined by crystal symmetry. An approximate (isotropic) treatment of cell e.s.d.'s is used for estimating e.s.d.'s involving l.s. planes.
Refinement. Refinement of *F*^2^ against ALL reflections. The weighted *R*-factor *wR* and goodness of fit *S* are based on *F*^2^, conventional *R*-factors *R* are based on *F*, with *F* set to zero for negative *F*^2^. The threshold expression of *F*^2^ > σ(*F*^2^) is used only for calculating *R*-factors(gt) *etc*. and is not relevant to the choice of reflections for refinement. *R*-factors based on *F*^2^ are statistically about twice as large as those based on *F*, and *R*- factors based on ALL data will be even larger.

### Fractional atomic coordinates and isotropic or equivalent isotropic displacement parameters (Å^2^)

**Table:**

	*x*	*y*	*z*	*U*_iso_*/*U*_eq_	
Br1	0.19197 (8)	0.40011 (8)	0.45593 (5)	0.0639 (3)	
O1	−0.2881 (7)	0.1341 (5)	0.3699 (4)	0.095 (2)	
C1	−0.6659 (9)	0.2445 (8)	0.6544 (5)	0.105 (4)	
H1A	−0.6484	0.3109	0.6864	0.158*	
H1B	−0.6483	0.1810	0.6897	0.158*	
H1C	−0.7597	0.2436	0.6295	0.158*	
Br2	−0.44399 (8)	0.39910 (9)	0.28265 (5)	0.0788 (4)	
O2	0.0549 (6)	0.6656 (5)	0.3651 (3)	0.0740 (18)	
C2	−0.5731 (9)	0.2412 (9)	0.5877 (5)	0.066 (3)	
C3	−0.5798 (8)	0.1529 (7)	0.5314 (5)	0.060 (2)	
H3A	−0.6427	0.0962	0.5349	0.072*	
C4	−0.4954 (8)	0.1491 (7)	0.4718 (5)	0.059 (2)	
H4A	−0.5023	0.0903	0.4347	0.071*	
C5	−0.3983 (7)	0.2326 (7)	0.4658 (4)	0.045 (2)	
C6	−0.3985 (8)	0.3206 (7)	0.5191 (5)	0.058 (2)	
H6A	−0.3403	0.3800	0.5138	0.070*	
C7	−0.4805 (10)	0.3237 (7)	0.5792 (5)	0.068 (3)	
H7A	−0.4738	0.3832	0.6156	0.081*	
C8	−0.3040 (8)	0.2209 (7)	0.4062 (5)	0.053 (2)	
C9	−0.2155 (7)	0.3172 (6)	0.3889 (4)	0.0425 (18)	
C10	−0.0778 (8)	0.3180 (6)	0.4244 (4)	0.0504 (19)	
H10A	−0.0436	0.2619	0.4603	0.061*	
C11	0.0067 (6)	0.4036 (6)	0.4052 (4)	0.0404 (17)	
C12	−0.0358 (7)	0.4850 (6)	0.3498 (4)	0.0422 (18)	
C13	−0.1739 (7)	0.4818 (6)	0.3136 (4)	0.0483 (19)	
H13A	−0.2061	0.5361	0.2755	0.058*	
C14	−0.2627 (7)	0.4003 (7)	0.3332 (4)	0.0464 (18)	
C15	0.0537 (8)	0.5788 (7)	0.3286 (4)	0.051 (2)	
C16	0.1406 (7)	0.5582 (7)	0.2634 (4)	0.0385 (18)	
C17	0.1434 (7)	0.4579 (7)	0.2242 (4)	0.051 (2)	
H17A	0.0876	0.4002	0.2374	0.062*	
C18	0.2315 (7)	0.4427 (7)	0.1637 (4)	0.050 (2)	
H18A	0.2341	0.3739	0.1383	0.060*	
C19	0.3118 (7)	0.5248 (8)	0.1418 (4)	0.053 (2)	
C20	0.3079 (8)	0.6253 (8)	0.1798 (5)	0.067 (3)	
H20A	0.3628	0.6828	0.1652	0.080*	
C21	0.2236 (8)	0.6427 (7)	0.2394 (5)	0.065 (2)	
H21A	0.2220	0.7120	0.2642	0.078*	
C22	0.4080 (8)	0.5080 (8)	0.0781 (4)	0.085 (3)	
H22A	0.3981	0.4339	0.0567	0.127*	
H22B	0.5006	0.5192	0.1033	0.127*	
H22C	0.3864	0.5600	0.0339	0.127*	

### Atomic displacement parameters (Å^2^)

**Table:**

	*U*^11^	*U*^22^	*U*^33^	*U*^12^	*U*^13^	*U*^23^
Br1	0.0470 (5)	0.0762 (6)	0.0651 (6)	−0.0002 (5)	−0.0061 (4)	0.0053 (5)
O1	0.116 (6)	0.083 (5)	0.098 (5)	−0.033 (4)	0.055 (4)	−0.022 (4)
C1	0.081 (7)	0.159 (11)	0.087 (7)	0.057 (7)	0.054 (6)	0.042 (7)
Br2	0.0463 (6)	0.1113 (8)	0.0740 (7)	−0.0026 (6)	−0.0098 (4)	0.0075 (6)
O2	0.091 (5)	0.074 (5)	0.064 (4)	−0.010 (4)	0.039 (3)	−0.018 (3)
C2	0.058 (6)	0.095 (8)	0.046 (5)	0.018 (5)	0.013 (5)	0.029 (6)
C3	0.045 (5)	0.065 (6)	0.072 (6)	−0.006 (4)	0.013 (5)	0.018 (5)
C4	0.054 (5)	0.076 (6)	0.049 (5)	0.005 (5)	0.010 (4)	0.011 (5)
C5	0.042 (5)	0.056 (5)	0.040 (5)	−0.009 (4)	0.012 (4)	0.002 (4)
C6	0.052 (5)	0.074 (7)	0.047 (5)	−0.013 (5)	0.002 (4)	−0.009 (5)
C7	0.094 (7)	0.062 (6)	0.053 (6)	0.004 (6)	0.033 (5)	0.006 (5)
C8	0.045 (5)	0.061 (6)	0.052 (5)	−0.003 (5)	0.008 (4)	−0.018 (5)
C9	0.046 (4)	0.051 (5)	0.035 (4)	−0.001 (4)	0.023 (3)	0.002 (3)
C10	0.063 (4)	0.051 (4)	0.040 (4)	0.005 (4)	0.015 (3)	0.008 (4)
C11	0.038 (4)	0.045 (4)	0.041 (4)	0.005 (3)	0.012 (3)	−0.003 (4)
C12	0.050 (4)	0.056 (5)	0.026 (4)	0.002 (4)	0.022 (3)	0.006 (3)
C13	0.054 (4)	0.058 (5)	0.032 (4)	0.010 (4)	0.000 (3)	0.014 (4)
C14	0.047 (4)	0.056 (4)	0.037 (4)	0.001 (4)	0.010 (3)	−0.001 (4)
C15	0.052 (5)	0.071 (7)	0.032 (5)	−0.005 (5)	0.011 (4)	−0.006 (4)
C16	0.034 (4)	0.058 (5)	0.025 (4)	0.005 (4)	0.008 (3)	0.001 (4)
C17	0.043 (5)	0.078 (6)	0.037 (5)	−0.001 (4)	0.023 (4)	−0.001 (4)
C18	0.046 (5)	0.050 (5)	0.055 (5)	0.005 (4)	0.007 (4)	0.001 (4)
C19	0.042 (5)	0.085 (7)	0.032 (5)	0.015 (5)	0.010 (4)	0.010 (5)
C20	0.063 (6)	0.083 (8)	0.056 (6)	−0.025 (5)	0.016 (5)	0.010 (5)
C21	0.065 (6)	0.074 (6)	0.060 (6)	−0.011 (5)	0.019 (5)	0.003 (5)
C22	0.070 (6)	0.136 (9)	0.054 (6)	−0.015 (6)	0.032 (5)	0.004 (6)

### Geometric parameters (Å, º)

**Table:**

Br1—C11	1.904 (6)	C10—C11	1.388 (9)
O1—C8	1.224 (8)	C10—H10A	0.9300
C1—C2	1.514 (10)	C11—C12	1.363 (9)
C1—H1A	0.9600	C12—C13	1.410 (9)
C1—H1B	0.9600	C12—C15	1.503 (9)
C1—H1C	0.9600	C13—C14	1.382 (9)
Br2—C14	1.867 (7)	C13—H13A	0.9300
O2—C15	1.204 (8)	C15—C16	1.475 (9)
C2—C7	1.369 (11)	C16—C17	1.371 (9)
C2—C3	1.403 (11)	C16—C21	1.395 (10)
C3—C4	1.363 (9)	C17—C18	1.411 (9)
C3—H3A	0.9300	C17—H17A	0.9300
C4—C5	1.402 (10)	C18—C19	1.346 (9)
C4—H4A	0.9300	C18—H18A	0.9300
C5—C6	1.374 (9)	C19—C20	1.365 (10)
C5—C8	1.439 (9)	C19—C22	1.512 (9)
C6—C7	1.352 (9)	C20—C21	1.378 (10)
C6—H6A	0.9300	C20—H20A	0.9300
C7—H7A	0.9300	C21—H21A	0.9300
C8—C9	1.502 (10)	C22—H22A	0.9600
C9—C14	1.391 (9)	C22—H22B	0.9600
C9—C10	1.404 (9)	C22—H22C	0.9600
			
C2—C1—H1A	109.5	C11—C12—C13	117.0 (7)
C2—C1—H1B	109.5	C11—C12—C15	123.8 (7)
H1A—C1—H1B	109.5	C13—C12—C15	119.1 (7)
C2—C1—H1C	109.5	C14—C13—C12	121.8 (7)
H1A—C1—H1C	109.5	C14—C13—H13A	119.1
H1B—C1—H1C	109.5	C12—C13—H13A	119.1
C7—C2—C3	117.3 (8)	C13—C14—C9	119.8 (6)
C7—C2—C1	121.9 (9)	C13—C14—Br2	120.1 (6)
C3—C2—C1	120.8 (9)	C9—C14—Br2	120.1 (6)
C4—C3—C2	120.9 (8)	O2—C15—C16	122.5 (7)
C4—C3—H3A	119.5	O2—C15—C12	120.5 (7)
C2—C3—H3A	119.5	C16—C15—C12	117.0 (7)
C3—C4—C5	120.9 (8)	C17—C16—C21	117.7 (7)
C3—C4—H4A	119.6	C17—C16—C15	122.4 (7)
C5—C4—H4A	119.6	C21—C16—C15	119.9 (7)
C6—C5—C4	116.9 (7)	C16—C17—C18	119.6 (7)
C6—C5—C8	124.1 (8)	C16—C17—H17A	120.2
C4—C5—C8	119.0 (8)	C18—C17—H17A	120.2
C7—C6—C5	122.2 (8)	C19—C18—C17	121.9 (8)
C7—C6—H6A	118.9	C19—C18—H18A	119.0
C5—C6—H6A	118.9	C17—C18—H18A	119.0
C6—C7—C2	121.6 (9)	C18—C19—C20	118.7 (7)
C6—C7—H7A	119.2	C18—C19—C22	121.9 (8)
C2—C7—H7A	119.2	C20—C19—C22	119.4 (9)
O1—C8—C5	123.2 (8)	C19—C20—C21	120.8 (8)
O1—C8—C9	117.1 (7)	C19—C20—H20A	119.6
C5—C8—C9	119.7 (7)	C21—C20—H20A	119.6
C14—C9—C10	119.1 (7)	C20—C21—C16	121.2 (8)
C14—C9—C8	121.9 (7)	C20—C21—H21A	119.4
C10—C9—C8	118.8 (7)	C16—C21—H21A	119.4
C11—C10—C9	119.2 (7)	C19—C22—H22A	109.5
C11—C10—H10A	120.4	C19—C22—H22B	109.5
C9—C10—H10A	120.4	H22A—C22—H22B	109.5
C12—C11—C10	123.1 (7)	C19—C22—H22C	109.5
C12—C11—Br1	119.9 (5)	H22A—C22—H22C	109.5
C10—C11—Br1	117.0 (6)	H22B—C22—H22C	109.5
			
C7—C2—C3—C4	1.1 (12)	C11—C12—C13—C14	0.1 (10)
C1—C2—C3—C4	−179.3 (7)	C15—C12—C13—C14	−177.3 (7)
C2—C3—C4—C5	0.8 (12)	C12—C13—C14—C9	−1.3 (11)
C3—C4—C5—C6	−3.8 (11)	C12—C13—C14—Br2	−179.4 (5)
C3—C4—C5—C8	175.1 (7)	C10—C9—C14—C13	0.1 (10)
C4—C5—C6—C7	5.0 (11)	C8—C9—C14—C13	−174.2 (7)
C8—C5—C6—C7	−173.8 (7)	C10—C9—C14—Br2	178.2 (5)
C5—C6—C7—C2	−3.2 (13)	C8—C9—C14—Br2	3.9 (9)
C3—C2—C7—C6	0.0 (12)	C11—C12—C15—O2	−92.6 (10)
C1—C2—C7—C6	−179.6 (8)	C13—C12—C15—O2	84.6 (9)
C6—C5—C8—O1	166.2 (8)	C11—C12—C15—C16	86.1 (8)
C4—C5—C8—O1	−12.7 (12)	C13—C12—C15—C16	−96.7 (8)
C6—C5—C8—C9	−11.4 (11)	O2—C15—C16—C17	177.2 (7)
C4—C5—C8—C9	169.8 (7)	C12—C15—C16—C17	−1.4 (10)
O1—C8—C9—C14	98.5 (9)	O2—C15—C16—C21	−2.9 (11)
C5—C8—C9—C14	−83.8 (9)	C12—C15—C16—C21	178.4 (7)
O1—C8—C9—C10	−75.8 (9)	C21—C16—C17—C18	1.8 (10)
C5—C8—C9—C10	101.9 (8)	C15—C16—C17—C18	−178.4 (6)
C14—C9—C10—C11	2.1 (10)	C16—C17—C18—C19	−1.3 (11)
C8—C9—C10—C11	176.6 (6)	C17—C18—C19—C20	0.4 (11)
C9—C10—C11—C12	−3.4 (10)	C17—C18—C19—C22	178.9 (7)
C9—C10—C11—Br1	179.7 (5)	C18—C19—C20—C21	0.0 (12)
C10—C11—C12—C13	2.2 (10)	C22—C19—C20—C21	−178.5 (7)
Br1—C11—C12—C13	179.1 (5)	C19—C20—C21—C16	0.5 (12)
C10—C11—C12—C15	179.5 (7)	C17—C16—C21—C20	−1.4 (11)
Br1—C11—C12—C15	−3.6 (9)	C15—C16—C21—C20	178.7 (7)
